# Integrated single-cell and bulk RNA sequencing reveals immune-related SPP1+ macrophages as a potential strategy for predicting the prognosis and treatment of liver fibrosis and hepatocellular carcinoma

**DOI:** 10.3389/fimmu.2024.1455383

**Published:** 2024-11-20

**Authors:** Bangjie Li, Jialiang Hu, Hanmei Xu

**Affiliations:** ^1^ Jiangsu Province Engineering Research Center of Synthetic Peptide Drug Discovery and Evaluation, China Pharmaceutical University, Nanjing, China; ^2^ State Key Laboratory of Natural Medicines, Ministry of Education, China Pharmaceutical University, Nanjing, China

**Keywords:** liver fibrosis, hepatocellular carcinoma, prediction model, SPP1, single cell sequencing

## Abstract

**Background:**

Liver fibrosis is a pathological response to liver damage induced by multiple etiologies including NASH and CCl_4_, which may further lead to cirrhosis and hepatocellular carcinoma (HCC). Despite the increasing understanding of liver fibrosis and HCC, clinical prognosis and targeted therapy remain challenging.

**Methods:**

This study integrated single-cell sequencing analysis, bulk sequencing analysis, and mouse models to identify highly expressed genes, cell subsets, and signaling pathways associated with liver fibrosis and HCC. Clinical prediction models and prognostic genes were established and verified through machine learning, survival analysis, as well as the utilization of clinical data and tissue samples from HCC patients. The expression heterogeneity of the core prognostic gene, along with its correlation with the tumor microenvironment and prognostic outcomes, was analyzed through single-cell analysis and immune infiltration analysis. In addition, the cAMP database and molecular docking techniques were employed to screen potential small molecule drugs for the treatment of liver fibrosis and HCC.

**Result:**

We identified 40 pathogenic genes, 15 critical cell subsets (especially Macrophages), and regulatory signaling pathways related to cell adhesion and the actin cytoskeleton that promote the development of liver fibrosis and HCC. In addition, 7 specific prognostic genes (CCR7, COL3A1, FMNL2, HP, PFN1, SPP1 and TENM4) were identified and evaluated, and expression heterogeneity of core gene SPP1 and its positive correlation with immune infiltration and prognostic development were interpreted. Moreover, 6 potential small molecule drugs for the treatment of liver fibrosis and HCC were provided.

**Conclusion:**

The comprehensive investigation, based on a bioinformatics and mouse model strategy, may identify pathogenic genes, cell subsets, regulatory mechanisms, prognostic genes, and potential small molecule drugs, thereby providing valuable insights into the clinical prognosis and targeted treatment of liver fibrosis and HCC.

## Introduction

Liver fibrosis and hepatocellular carcinoma are two critical stages in the development of liver disease and are closely related. Hepatic fibrosis, a pathological response to liver injury caused by multiple etiologies (including NASH and CCl_4_), is characterized by abnormal proliferation of fibrous tissue and excessive collagen deposition in the liver due to an imbalance between increased synthesis and insufficient degradation of extracellular matrix (ECM) (1–3). Although there may be no obvious symptoms in the early stage of liver fibrosis except fatigue, loss of appetite and abdominal pain, with the increase of fibrous tissue, the structure and function of the liver will be gradually damaged, leading to cirrhosis and hepatocellular carcinoma (HCC) (4–6). Currently, HCC remains one of the most common malignancies in the world and the fourth leading cause of cancer-related death, with an approximately 20% 5-year survival rate (7, 8). However, the early diagnosis rate of HCC is extremely low due to the rapid growth and inconspicuous early symptoms (except weight loss and abdominal pain), and most cases are already in the middle and late stages when clinically diagnosed (9, 10).

The current diagnostic methods for HCC mainly comprise serum tumor marker detection (alpha fetoprotein, AFP), imaging examination including ultrasound, computed tomography (CT), magnetic resonance imaging (MRI), and positron emission tomography/computed tomography (PET/CT), which are the most commonly used methods in clinical practice due to their convenience, real-time and noninvasive characteristics. In addition, invasive liver biopsy is still widely regarded as the gold standard for diagnosis, significantly improving the prognosis and survival rate of patients (11–13). However, the existing diagnostic methods still have limitations in detecting early HCC. For instance, alpha-fetoprotein (AFP), a common diagnostic marker for HCC, does not always provide satisfactory diagnostic accuracy. Moreover, although CT, MRI and other imaging techniques play an important role in the diagnosis of HCC, their detection ability for small hepatocellular carcinoma (less than 5 cm in diameter) is limited. In addition, the spatial resolution and contrast of these technologies are also limited, which may not accurately identify the subtle structures of tumors. More importantly, individual differences and the complexity of HCC, as well as the influence of tumor microenvironment, will lead to increased difficulty in diagnosis and improper treatment (14–16). At present, the HCC therapies mainly consist of surgical therapy (liver resection and transplantation), local ablation, interventional therapy (transcatheter arterial chemoembolization (TACE)), radiotherapy, and systematic drug therapy including targeted therapy, immunotherapy and chemotherapy, and other complementary therapies. Among them, local therapies such as surgical resection and ablation are considered the most effective treatment for early HCC, but only less than 30% of HCC patients are eligible for this treatment. Interventional therapy is the main treatment for intermediate HCC, but the prognosis of advanced HCC patients after treatment is still unsatisfactory (17, 18). Moreover, the frequent recurrence and metastasis after traditional treatments such as surgery and chemotherapy often lead to poor prognosis for patients (19, 20). In recent years, breakthrough progresses have been made in systematic drug therapy suitable for patients with advanced HCC, such as targeted drugs (e.g. Sorafenib, Lenvatinib, Apatinib and Bevacizumab) and immune drugs (e.g. Nivolumab, Pembrolizumab, Sintilimab, and Atezolizumab), which have improved the efficacy and prolonged the survival of patients. However, single drug therapy is no longer sufficient to meet the clinical treatment needs of advanced HCC, especially after significant progress was made in the global multicenter Phase III study called ImBrave 150 (Atezolizumab+Bevacizumab, ATZ+BEV). Significantly, in this clinical trial, the median overall survival (mOS) of patients increased to 19.2 months and the objective remission rate (ORR) reached 30%, making targeted combined immunotherapy a new standard for the first-line treatment of advanced HCC (21–23). In summary, although the existing diagnostic and therapeutic methods have played important roles in the management of HCC, there are still many limitations and efforts should be made to improve the accuracy, sensitivity and specificity of diagnosis, as well as develop more personalized diagnosis and treatment strategies.

Several previous studies have analyzed some genetic factors that cause liver fibrosis or HCC, but there is a lack of specific research on pathogenic genes, cell subpopulations and signaling pathways, which may result in the failure of early detection and thus the inability to successfully intervene in the clinic (24–26). Therefore, it is urgent to identify novel targets for the effective prediction and targeted treatment of liver fibrosis and HCC. In the past decade, the rapid development of deep sequencing technology and bioinformatics technology has provided excellent opportunities for analyzing the immunological characteristics, potential biomarkers and therapeutic drugs of diseases at the single-cell level (27–29).

This study integrated single-cell sequencing datasets of NASH and CCl_4_-induced liver fibrosis, as well as bulk sequencing data of independently constructed CCl_4_-induced mouse liver fibrosis to screen out 40 pathogenic genes and their expression distribution in different cell subpopulations, and identified cell adhesion and actin cytoskeleton regulatory signaling pathways that may promote the development of liver fibrosis. In addition, a clinical prediction model was established and validated through machine learning, survival analysis and clinical samples, thereby identifying a core prognostic gene SPP1, and its expression heterogeneity between liver fibrosis and HCC and positive correlation with immune infiltration and prognostic development were also interpreted. Finally, six potential small molecule drugs for liver fibrosis and HCC were screened using the cMAP database and molecular docking, with the aim of achieving effective prognosis and targeted treatment for patients with liver fibrosis and HCC.

## Materials and methods

### The single cell sequencing data download and processing

The single cell sequencing data of human non-alcoholic steatohepatitis (NASH)-induced liver fibrosis, CCl_4_-treated mouse liver fibrosis, and hepatocellular carcinoma patient samples (specifically, GSE212837, GSE132662 and GSE242889) were downloaded from the NCBI database (https://www.ncbi.nlm.nih.gov/). Additionally, liver cancer datasets for external validation of the model were acquired from ICGC Data Portal (https://daco.icgc-argo.org/, a valid Google email address, such as Gmail or G Suite, is required for logging in and obtaining ICGC controlled data access authorization). All sequencing reads were mapped to the human and mouse reference genome (GRCh38, GRCm38) and genome annotations (GRCh38.84.gtf, GRCm38.102.gtf) sourced from the Ensembl database (https://asia.ensembl.org/). Subsequent downstream analysis was conducted using Cell ranger (30).

### Visualization analysis of cell subpopulations and differentially expressed genes

Firstly, quality control of the single-cell sequencing data was performed using the parameters set in the Scanpy software developed by the 10x Genomics company. Cells were retained if they had genes expressed in at least 3 cells and more than 200 genes expressed per cell, as implemented by functions such as sc.pp.filter_cells(adata, min_genes=200) and sc.pp.filter_genes(adata, min_cells=3). Additionally, cell filtering was also based on the number of genes detected in each cell and the proportion of mitochondrial gene expression relative to the total gene expression levels in the corresponding sequencing results. Specifically, for the NASH-derived data, the filtering parameters were set to retain cells with fewer than 4500 genes detected (adata = adata[adata.obs[‘n_genes_by_counts’] < 4500],): and a mitochondrial gene expression proportion of less than 25% (adata = adata[adata.obs[‘pct_counts_mt’] < 25],):. For the CCl_4_-derived data, the filtering parameters were adjusted to retain cells with fewer than 6000 genes detected (adata = adata[adata.obs[‘n_genes_by_counts’] < 6000],): and a mitochondrial gene expression proportion of less than 20% (adata = adata[adata.obs[‘pct_counts_mt’] < 20],):. Following these filtering steps, data normalization analysis was conducted. The single-cell gene expression data were then dimensionally reduced and clustered, with cell types annotated using CellTypist (31). Trajectory analysis and differential gene identification were subsequently performed using SCANPY software (32).

### Establishment of liver fibrosis model

Before conducting the liver fibrosis model experiments, 30 male C57BL/6 mice (weighting 20-22 g and aged 6-8 weeks), obtained from Nanjing Cavens Biotechnology Co., Ltd (Contract number: 2020112508), were acclimatized for a week in Specific Pathogen Free (SPF) conditions at the Pharmaceutical Animal Experiment Center of China Pharmaceutical University. The mice were divided into a model group and a control group, with the model group receiving intraperitoneal injections of CCl_4_+mineral oil for 8 weeks and the control group receiving physiological saline for the same time duration (33, 34). All animal experiments were conducted in accordance with the protocols approved by the Institutional Animal Care and Use Committee of China Pharmaceutical University and were also approved by the Ethics Committee of China Pharmaceutical University (Permit Number SYXK2012-0035).

### Histopathological assessment

Histopathological assessment was conducted by Hematoxylin–Eosin (H&E) staining and Masson’s trichrome staining. Briefly, the mice’s hepatic tissues were fixed by 4% paraformaldehyde solution, followed by gradient ethanol dehydration and embedding in paraffin (35). The processed tissues were then cut into 5-micrometer-thick sections, dewaxed, and stained with the respective dyes for observation under an optical microscope (Olympus Co., Ltd., Tokyo, Japan).

### Serum levels detection of ALT and AST

Serum samples were obtained from the control group and liver fibrosis model group of mice. Subsequently, the levels of alanine aminotransferase (ALT) and aspartate aminotransferase (AST) were measured using corresponding assay kits provided by Jiancheng Co., Ltd. (Nanjing, China), ac-cording to the manufacturer’s protocol.

### Stranded transcriptome sequencing

Liver fibrosis tissues were collected from the mice, and total RNA was extracted using a TRIzol reagent kit (Thermofisher Co., Ltd., California, USA) according to the manufacturer’s protocol. The RNA concentration and band quality were tested via NanoDrop 2000 spectrophotometer and agarose gel electrophoresis, respectively. Qualified mRNA was then enriched, fragmented, reverse-transcribed, purified, and sequenced using Illumina Novaseq 6000 sequencing platform by a commercial biotechnology corporation (Gene Denovo Co., Ltd., Guangzhou, China).

### RNA extraction and real-time quantitative PCR

Hepatic stellate cells (HSCs) and LX-2 were collected from both the control group and the liver fibrosis model group. Total RNA was extracted from these cells using a TRIzol reagent kit (Invitrogen, Carlsbad, USA) according to the manufacturer’s protocol. The extracted RNA was then used to synthesize cDNA with the HiScript III RT SuperMix for qPCR kit (Vazyme, Nanjing, China). Real-time PCR detection was conducted using ChamQ SYBR qPCR Master Mix (Vazyme, Nanjing, China). GAPDH was used as the internal control gene. The primer sequences are provided in **Supplementary Table 1**.

### Transcriptome and clinical data analysis of LIHC in TCGA database

The transcriptional expression profiles (n= 424) and clinical data (n= 377) of patients with liver hepatocellular carcinoma (LIHC) were downloaded from the TCGA database using the R package TCGAbiolinks (36). Data extraction, classification, and differential gene analysis were performed using TCGAbiolinks, DESeq2, edgeR and limma (37–39), respectively. In the differential gene expression (DEG) analysis, all genes with adjusted P values less than 0.05 and absolute log2 fold change (log2FC) values greater than 1 were considered statistically significant.

### Construction and validation of the LIHC−related prognostic signature

The genes highly expressed in both liver fibrosis and HCC were selected by three methods: single-cell data analysis, transcriptome data analysis, and mouse model construction. To further investigate the correlation and importance of these genes with patient survival time, only the corresponding patient survival datasets extracted from the TCGA database were used to construct a univariate cox proportional hazard model using the survival R package and the coxph function. In the process of building the prediction model for this study, we applied a random splitting method to divided the extracted HCC patient dataset from the TCGA database into training and testing datasets in a 7:3 ratio. The specific code is as follows: library(caret), set.seed(100), index <- createDataPartition(y = exp$ID, p = 0.7, list = FALSE), train.data <- exp[index], test.data <- exp[-index],. The filtered variables were then subjected to Least Absolute Shrinkage and Selection Operator (LASSO) analysis using the glmnet R package, and candidate genes were identified for a multivariate Cox proportional hazard model based on the optimal penalty parameter λ determined by lambda.min. Furthermore, to evaluate the performance of the constructed model, an independent clinical cohort dataset of liver cancer patients, known as LIRI-JP, was processed from ICGC Data Portal for external verification of the model. Forest plot of candidate differentially expressed genes and the nomogram plot of significant variables were drawn using the survminer R package and the rms R package to visualize their close relationship with the development and prognosis of HCC. To evaluate the model’s performance, significant variables were added to the constructed model, and a calibration curve was drawn to compare predicted and actual values. Furthermore, HCC patients were stratified into high-risk or low-risk groups based on the median risk score, and the predictive performance of the prognostic model was evaluated using Kaplan-Meier survival analysis and time-dependent ROC analysis. High- and low-risk heatmaps of the prognostic model were drawn using the tidyverse R package and the pheatmap R package.

### Verification of protein expression level of hub gene in LIHC

Immunohistochemistry (IHC) images, recorded in the Human Protein Atlas database (https://www.proteinatlas.org/), were used to verify the protein expression of specific hub genes in LIHC and normal tissues, both detected using the same antibody (HPA027541).

### Immune cell infiltration analysis

Using the CIBERSORT R package, immune cell infiltration analysis on the differentially expressed genes derived from the mouse liver fibrosis model was conducted, aiming to identify the immune cell types that showed significant differences between liver fibrosis and normal samples (40). Additionally, the TIMER2 database provides a data resource for quantifying immune cell infiltration levels across different cancer types (41). Within the TCGA database, the correlations between SPP1 expression and 14 immune cell subsets, including B cells, myeloid dendritic cells, cancer-associated fibroblasts, macrophages (M0, M1, M2), macrophages/monocytes, monocytes, neutrophils, activated NK cells, CD4+Th2 T cells, regulatory T cells (Tregs), and myeloid-derived suppressor cells (MDSCs) in LIHC, were analyzed using purity-adjusted Spearman correlation analysis.

### Connectivity map analysis and molecular docking

The CMAP database, the world’s largest gene expression profile database, is based on perturbations of hundreds of tumors and normal cell lines from various tissue sources. It includes approximately 3000 small molecules with clear mechanisms of action (MOA), affected pathways, and target proteins, revealing relationships between diseases, genes and drugs (42, 43). The normalized connectivity score (NCS) represents the enrichment of transcriptional differences after perturbations, with negative values indicating opposite directions of transcriptional regulation. Therefore, 40 highly expressed genes were uploaded into the cMAP database to search for potential small molecule drugs for the treatment of liver fibrosis and HCC. Subsequently, the normalized connectivity score (NCS) was sorted in ascending order and all compounds with NCS less than -2 were screened. The three-dimensional structures of these compounds were downloaded from the PubChem database (https://pubchem.ncbi.nlm.nih.gov/) and used as ligands for molecular docking with the screened hub protein through the CB-Dock2 server (44). Finally, their binding sites and affinities were predicted to identify potential small molecule drugs for the treatment of liver fibrosis and HCC.

### Quantification and statistical analysis

All data presented in this study are shown as mean ± SEM from at least three independent experiments. Significance was determined using Student’s t-test conducted with GraphPad Prism 9.5 software. A P-value of less than 0.05 was considered statistically significant.

## Results

### Single-cell analysis of liver fibrosis

**Figure 1:**
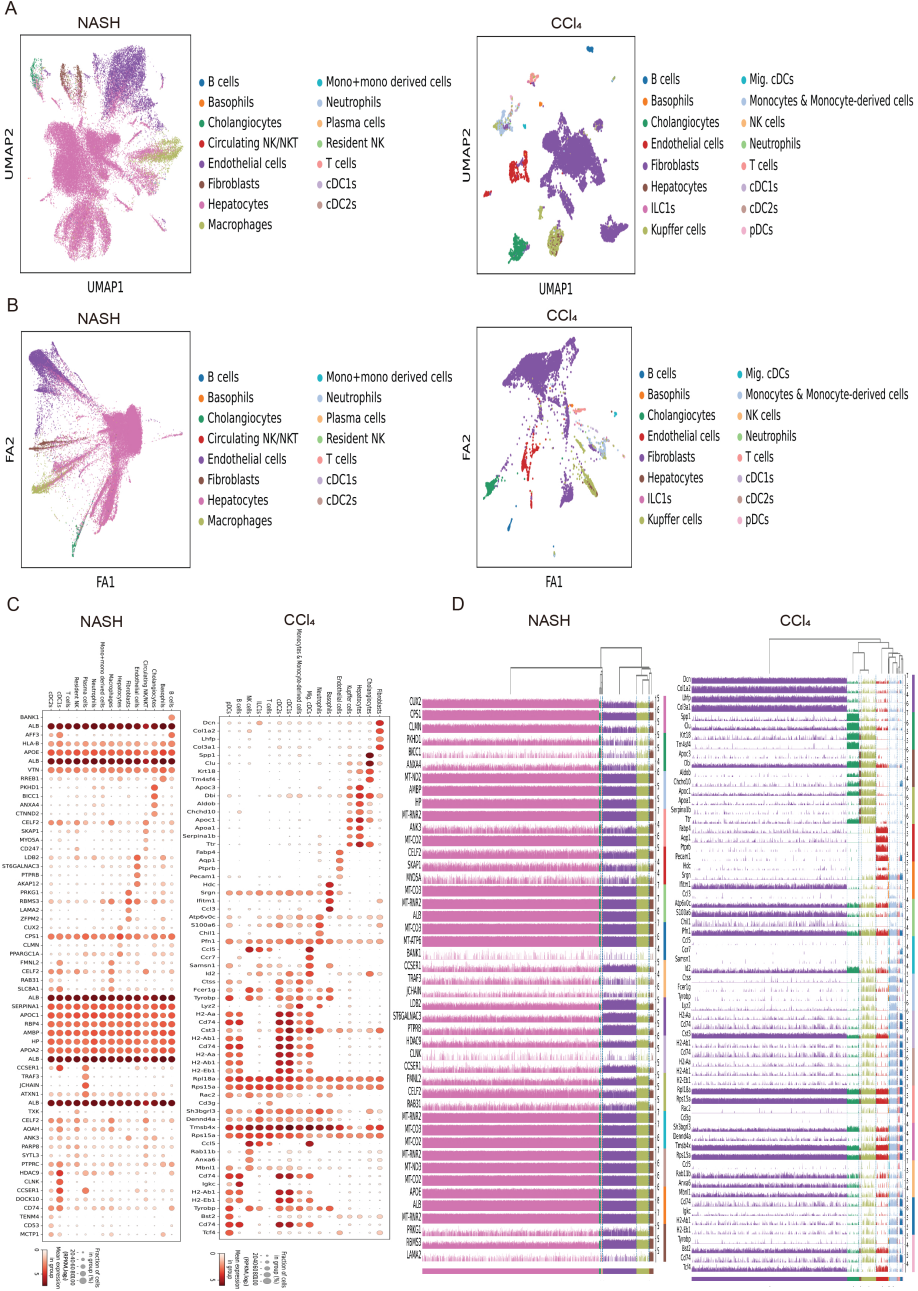
Expression distribution and trajectory analysis of highly expressed genes in human and mouse liver fibrosis cell subpopulations. **(A)** Visual analysis of cell sub-populations and cell type annotations in the human NASH-induced and CCl_4_-treated liver fibrosis. **(B)** The cell trajectory analysis over time of 15 human NASH-induced cell clusters (left) and 16 mouse CCl_4_-treated cell clusters (right). **(C)** Dot plots of genes highly expressed in human NASH-induced (left) and CCl_4_-treated (right) liver fibrosis cell subpopulations. **(D)** Tracksplot of gene expression values in different cell subsets in 15 human NASH-induced (left) and 16 mouse CCl_4_-treated (right) liver fibrosis.

To explore the distribution types of cell subpopulations among differentially expressed genes, cell type annotation was applied to visually analyze human and mouse single cell samples. 15 human cell subpopulations (Hepatocytes, Cholangiocytes, Neutrophils, T cells, Circulating NK/NKT, Resident NK, B cells, Plasma cells, Endothelial cells, cDC1s, Macrophages, Mono+monocyte-derived cells, cDC2s, Basophils, Fibroblasts) and 16 mouse cell subpopulations (Fibroblasts, Cholangiocytes, Hepatocytes, Kupffer cells, Endothelial cells, Basophils, Neutrophils, Migratory cDCs, Monocytes & Monocyte-derived cells, cDC1s, cDC2s, T cells, ILC1s, NK cells, B cells, pDCs) were identified (**Figure 1A**). The results revealed that hepatocytes and endothelial cells were the predominant cell subpopulations in patients with non-alcoholic steatohepatitis (NASH), whereas fibroblasts were the main cell subpopulation in the livers of CCl_4_-treated mice liver fibrosis. To infer the evolution and differentiation processes between different cell types at the single-cell level by constructing the trajectory of cellular changes over time, fifteen human and sixteen mouse cell clusters were obtained based on cell dimensionality reduction and type clustering (**Figure 1B**). Furthermore, 60 human and 64 mouse highly expressed genes in cell subpopulations were filtered out (**Figure 1C**), and gene expression values in different cell subsets in 15 human NASH-induced and 16 mouse CCl_4_-treated liver fibrosis were also obtained (**Figure 1D**).

### Establishment of liver fibrosis model

**Figure 2:**
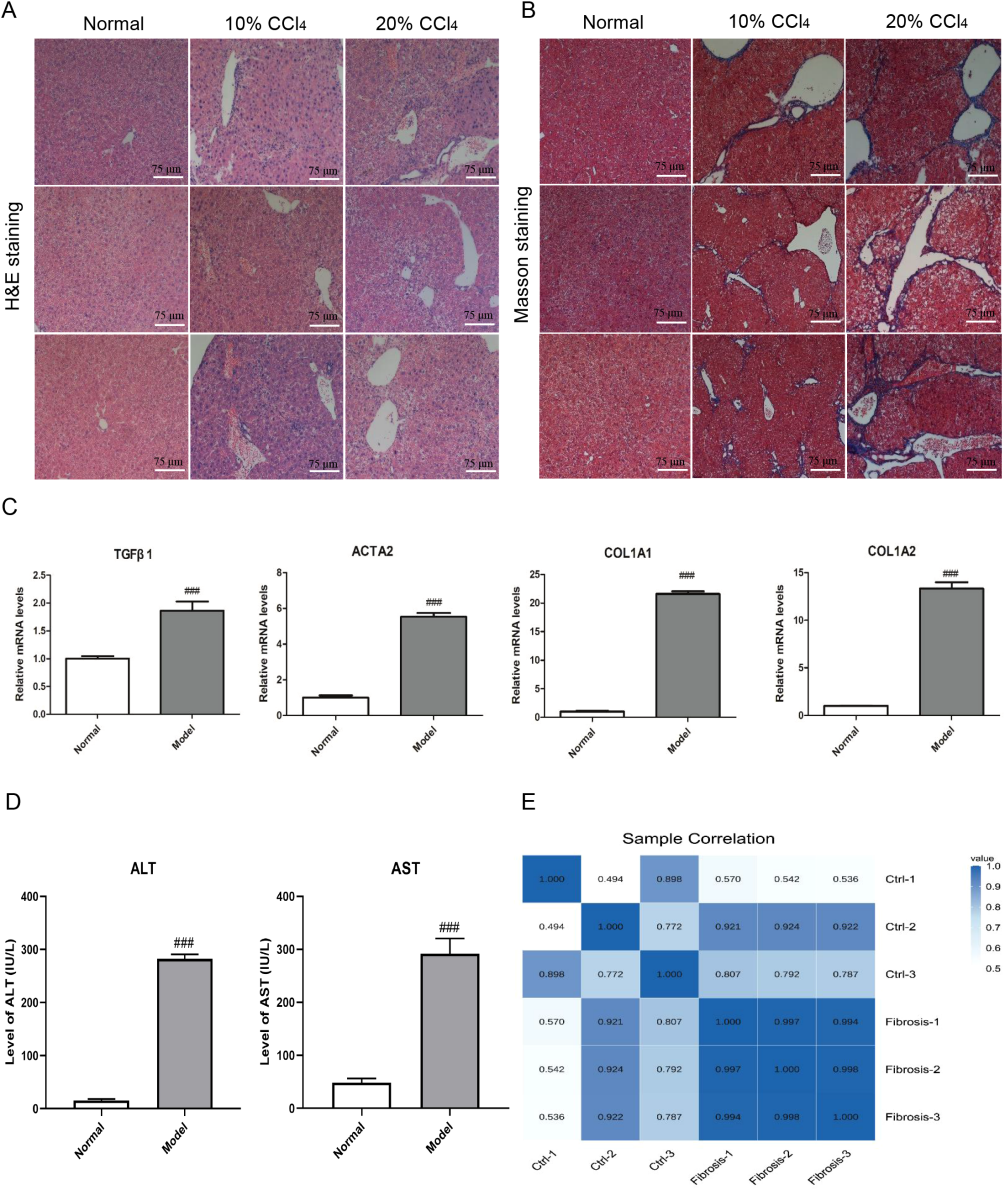
Histopathological evaluation and biomarker detection of liver fibrosis in mice. **(A)** Histopathological assessment of hematoxylin–eosin of mouse liver tissues in normal and fibrosis model groups. Each column represents the histological morphology of three different mice under the same treatment condition. Scale bar =75 μm. **(B)** Histopathological assessment of masson staining of mouse liver tissues in normal and fibrosis model groups. Each column represents the histological morphology of three different mice under the same treatment condition. Scale bar = 75 μm. **(C)** The mRNA expression detection of 4 representative fibrosis markers of mouse liver tissues in normal and fibrosis model groups. A total of 6 samples and each experiment was repeated three times. The 2^−ΔΔCt^ method was used to analyze the relative expression of target gene mRNA. Data was presented as mean ± S.D and the significance of differences between the Normal and Model groups was evaluated using the Student’s t-tests using GraphPad Prism 9.5 software. ###p<0.001 were considered statistically significant. **(D)** The serum ALT and AST levels detection in normal and fibrosis model groups. A total of 6 samples and each experiment was repeated three times. ###p<0.001 were considered statistically significant. **(E)** Pearson correlation coefficient heatmap of the expression levels between mouse normal and fibrosis liver tissue samples.

Histopathological assessment of mouse liver tissues was conducted using H&E and Masson staining (**Figures 2A, B**). The experimental results indicated that, compared to the control group, the collagen fibers in the liver tissues of the fibrosis group were significantly increased. Furthermore, the gene expression levels of some representative fibrotic markers, as well as the serum levels of ALT and AST, were higher in the liver fibrosis model group than in the normal group (**Figures 2C, D**). Additionally, the Pearson correlation coefficient heatmap of the expression levels in liver tissue samples prepared for subsequent sequencing also revealed significant differences between liver fibrosis and normal liver tissue samples, and demonstrated good reproducibility among liver fibrosis samples (**Figure 2E**).

### Functional enrichment analysis of DEGs in mouse hepatic fibrosis

**Figure 3:**
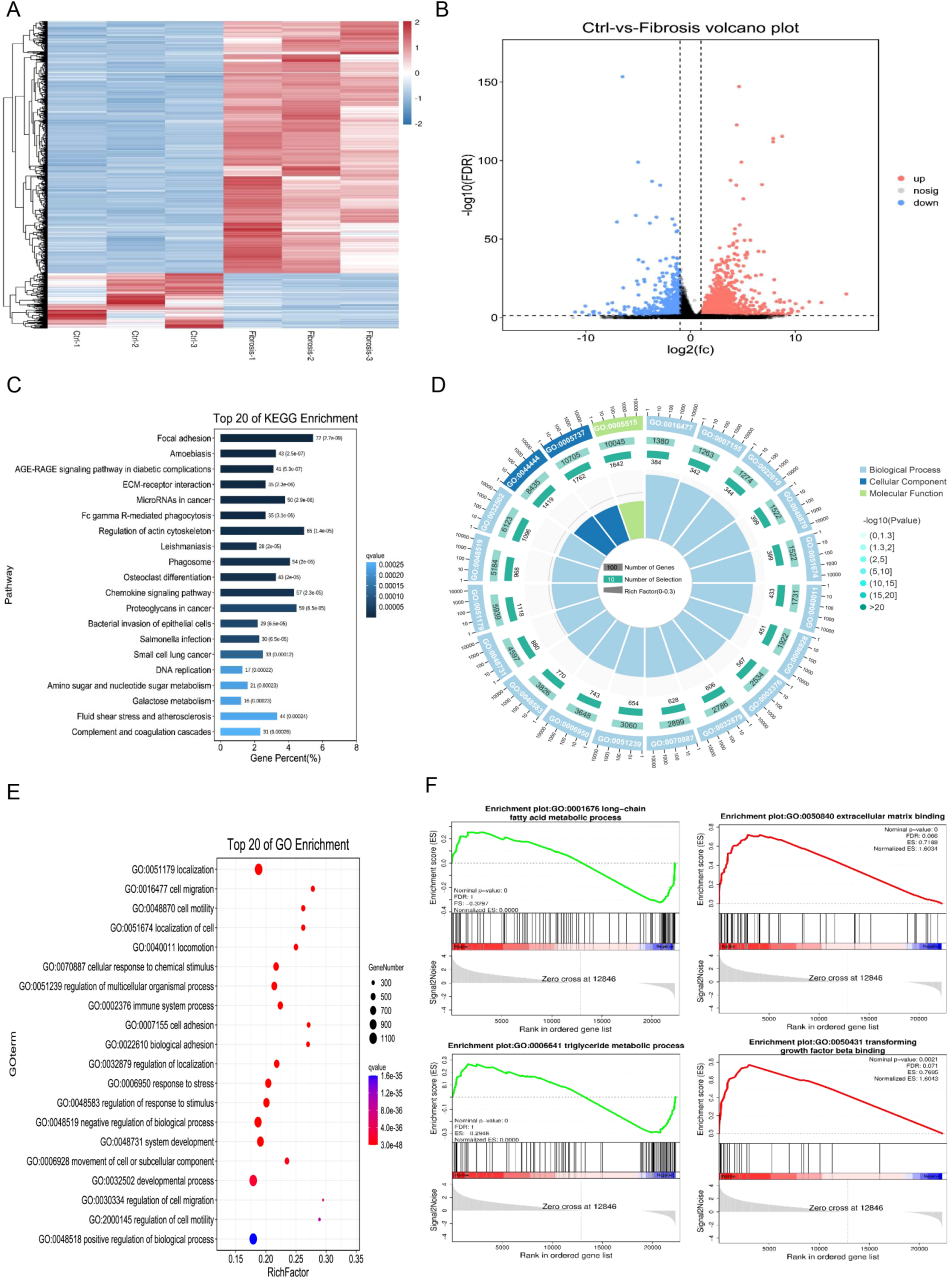
Functional enrichment analysis of differentially expressed genes in mouse hepatic fibrosis. **(A)** Heatmap for hierarchical clustering of differential gene expression patterns between control and liver fibrosis groups. **(B)** Volcano plot of significant gene expression differences between control and fibrosis groups, including 2328 up-regulated genes and 509 down-regulated genes. **(C)** Top 20 enriched KEGG pathways in differential gene analysis of RNA sequencing data of normal and fibrosis mouse liver tissues. **(D)** GO annotation of differential genes between normal and fibrosis mouse liver tissues, including biological processes (BP), cellular components (CC), and molecular functions (MF). **(E)** Top 20 enriched GO pathways in differential gene analysis of RNA sequencing data of normal and fibrosis mouse liver tissues. **(F)** Gene Set Enrichment Analysis (GESA) of differential genes in normal and fibrosis mouse liver tissues.

The expression patterns of differentially expressed genes between the control group and the liver fibrosis group were analyzed using hierarchical clustering analysis, and the results showed that liver fibrosis significantly affects the expression patterns (**Figure 3A**). There were significant differences in bulking sequencing of mouse liver fibrosis group, including 2328 upregulated genes and 509 downregulated genes (**Figure 3B**). Based on KEGG, Reactome and GO database, the pathway and GO (Gene Ontology) analysis of differential genes showed that focal adhesion and regulation of actin cytoskeleton signaling pathways were the most significantly enriched, and mainly participated in localization, cell adhesion and system development that occur in the intracellular part, actin cytoskeleton and cytoplasm (**Figures 3C–E**). The Gene Set Enrichment Analysis (GESA) from the GO and Molecular Signatures Database (MSigDB, https://www.gsea-msigdb.org/gsea/msigdb) also suggested that most differentially genes were closely related to long-chain fatty acid metabolic process, triglyceride metabolic process, extracellular matrix binding and transforming growth factor beta binding in hepatic fibrosis (**Figure 3F**).

### The immune infiltration analysis of DEGs in liver fibrosis

**Figure 4:**
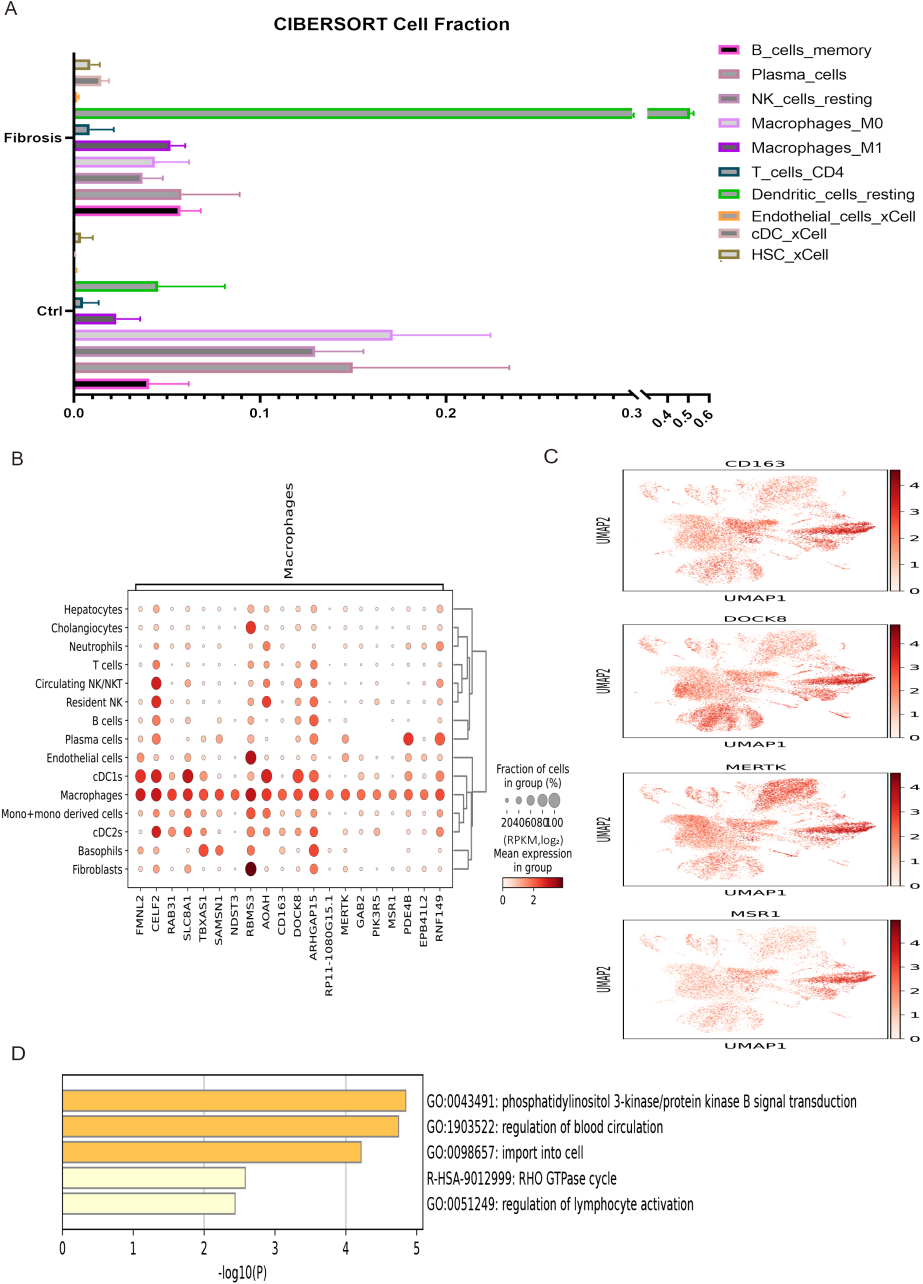
The immune infiltration of liver fibrosis and enrichment analysis of macrophages. **(A)** The immune infiltration analysis of 10 cell subpopulations between control and liver fibrosis. **(B)** Top 20 highly expressed genes in macrophages subpopulation of liver fibrosis. **(C)** The expression distribution of 4 representative highly expressed genes in macrophages subpopulations. **(D)** The enrichment analysis of DEGs in macrophages subpopulation.

To analyze the regulatory effects of immune cells on liver fibrosis development, immune infiltration analysis was performed on differentially expressed genes between mouse liver fibrosis and a control group (**Figure 4A**). The results of this analysis revealed that liver fibrosis significantly affected 10 cell subpopulations, particularly macrophages. The top 20 highly expressed genes in macrophages from human fibrosis single-cell sequencing were screened and the expression distribution of four marker genes was displayed, which indicated that macrophages were markedly activated during the progression of liver fibrosis (**Figures 4B, C**). To further elucidate the impact of macrophages on liver fibrosis, enrichment analysis was conducted on these highly expressed genes (**Figure 4D**).

### Comparison of DEGs between single-cell sequencing and bulk sequencing

**Figure 5:**
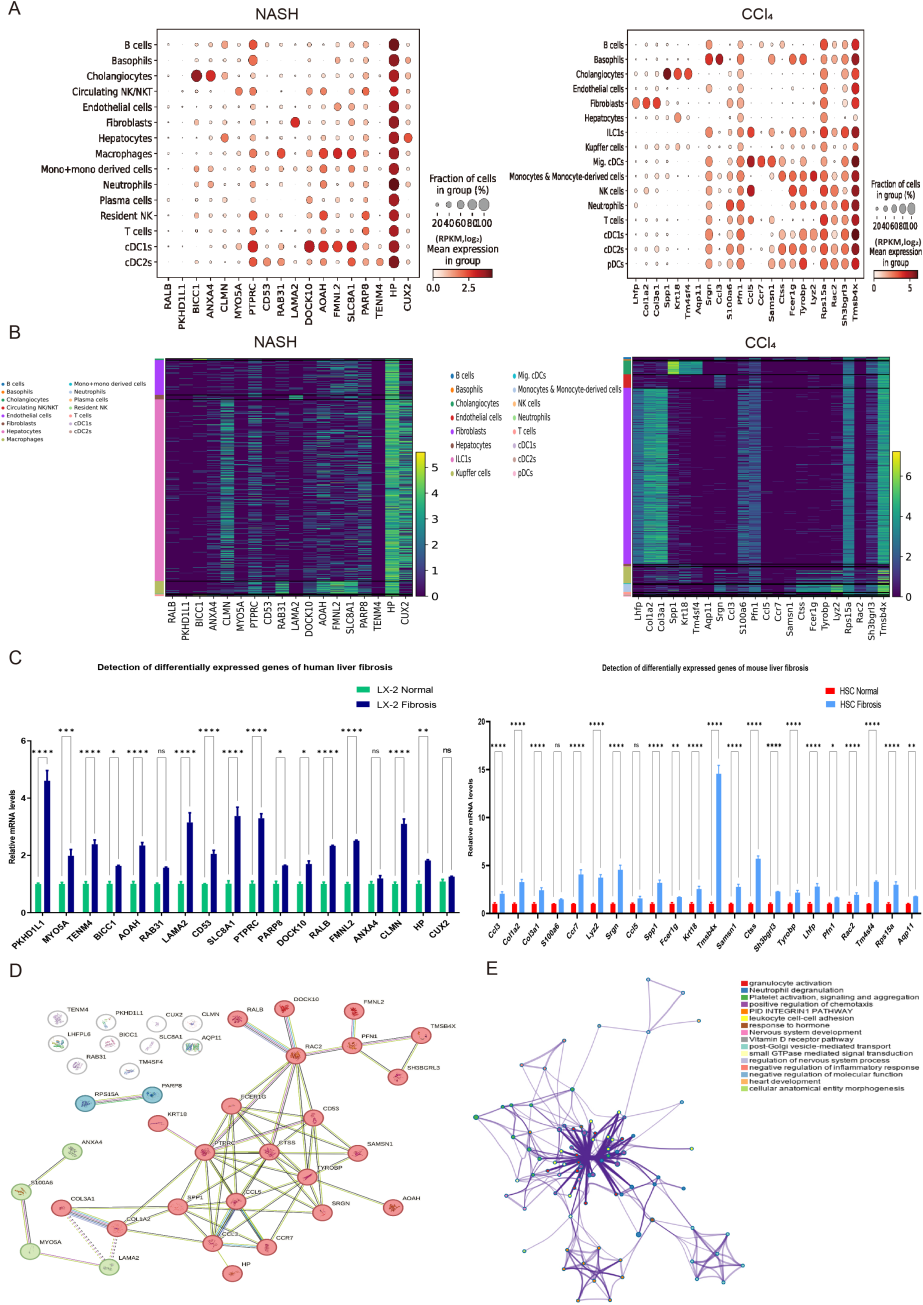
The cell subpopulation distribution, experimental verification of expression levels, and enrichment analysis of 40 liver fibrosis related DEGs. **(A)** Dot plot analysis of 18 human and 22 mouse differentially expressed genes (DEGs) in cell subpopulations of human NASH-induced and mouse CCl_4_-treated liver fibrosis. **(B)** Expression heatmap of 18 human and 22 mouse DEGs in cell subpopulations of human NASH-induced and mouse CCl_4_-treated liver fibrosis. **(C)** Gene expression detection of were detected in human and mouse hepatic stellate cells by RT-qPCR analysis. A total of 12 samples and each experiment was repeated three times. The 2^−ΔΔCt^ method was used to analyze relative expression of target gene mRNA. Data was presented as mean ± S.D and the significance of differences between the Normal and Fibrosis groups was evaluated using the Student’s t-tests using GraphPad Prism 9.5 software. *p<0.05, **p<0.01, ***p<0.001 or ****p<0.0001 were considered statistically significant. **(D, E)** The protein–protein interaction (PPI) network and pathway enrichment analysis of 40 liver fibrosis related DEGs.

The 60 human and 64 mouse differentially expressed genes (DEGs) from single-cell data were compared with 2837 DEGs in mouse liver fibrosis. As a result, 18 human and 22 mouse genes were found to be significantly different in both single-cell and bulk sequencing analyses. Furthermore, the expression distribution of these 18 human and 22 mouse DEGs was analyzed across various subgroups in the human and mouse single-cell sequencing datasets (**Figures 5A, B**).

### Experimental verification and enrichment analysis of 40 DEGs

To verify the differential expression levels of these 40 screened genes, which included 18 human and 22 mouse differentially expressed genes (DEGs), RT-qPCR analysis was conducted using cultured hepatic stellate cells (**Figure 5C**). Additionally, protein-protein interaction and pathway enrichment analyses were carried out on the 40 DEGs. The results revealed that protein interaction network could be categorized into three clusters, with the largest cluster of genes potentially playing a major role in the development of liver fibrosis (**Figure 5D**). Furthermore, cell adhesion was found to be significantly regulated and closely associated with cell activation, signal transduction and systemic development (**Figure 5E**). These findings were consistent with the enrichment results obtained from bulk sequencing of mouse liver fibrosis.

### Construction and validation of a prognostic model for HCC

**Figure 6:**
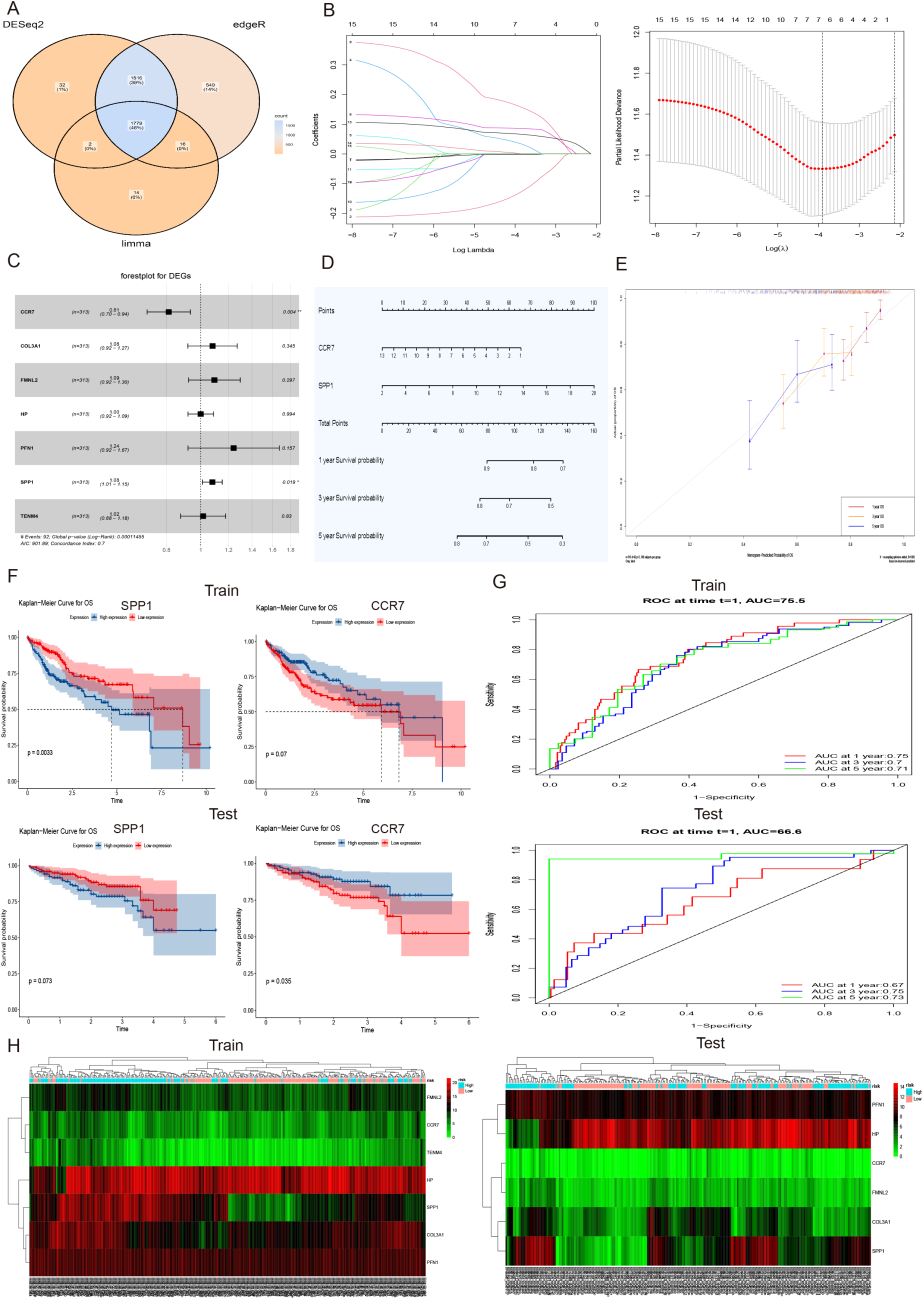
Construction and validation of a prognostic model for HCC. **(A)** Venn diagram of 1779 upregulated differentially expressed genes (DEGs) in transcriptome data of patients (n = 424) in TCGA-LIHC dataset using three algorithms: DESeq2, edgeR, and limma. **(B)** LASSO coefficient profiles of 40 screened liver fibrosis and HCC related DEGs (Left), and tenfold cross validation of the LASSO model, with vertical dashed lines are plotted at the minimum deviance (log(l.min)) and 1 standard error of the minimum deviance (log(l.1se)) (Right). **(C)** The forest plots of 7 specific genes, including CCR7, COL3A1, FMNL2, HP, PFN1, SPP1 and TENM4. The significance of differences was evaluated, *p<0.05, **p<0.01 were considered statistically significant. **(D)** The nomogram plot of significant variables CCR7 and SPP1 with the 1, 3, and 5-year survival of LIHC. **(E)** The calibration curves for survival at 1, 3, and 5-year intervals displayed high consistency with the expected survival probability. **(F)** Kaplan-Meier survival analysis of the prognostic value of CCR7 and SPP1 in the train and test HCC patient cohort. The differences for CCR7, p = 0.07 and SPP1, p = 0.0033 in the train cohort, and significance of test cohort was CCR7, p = 0.035 and SPP1, p = 0.073, respectively. **(G)** The receiver operating characteristic (ROC) investigation of model performance, and the area under curve (AUC) values of train cohort were 0.75, 0.70, and 0.71 and area values of test cohort were 0.67, 0.75 and 0.73 for predicting 1, 3, and 5 years, respectively. **(H)** The heatmap of the expression levels of 7 specific genes in train and test HCC patient cohort with high and low risk scores.

The transcriptome data of patients (n = 424) from the TCGA-LIHC dataset was analyzed using three algorithms: DESeq2, edgeR, and limma for differential gene expression. This analysis yielded 1779 common up-regulated genes (**Figure 6A**), among which 40 genes related to liver fibrosis were screened. Subsequently, a univariate Cox proportional hazard model, Lasso regression and multivariable Cox regression were applied to these 40 screened genes along with the corresponding survival data of liver cancer patients, identifying 7 specific genes (CCR7, COL3A1, FMNL2, HP, PFN1, SPP1 and TENM4) that were associated with survival outcomes (**Figure 6B**). Furthermore, the forest plots of these specific genes and a nomogram plot of significant variables visually demonstrated the significant associations of CCR7 and SPP1 with 1, 3, and 5-year survival of LIHC (**Figures 6C, D**). To evaluate the model’s performance, calibration curves for survival at 1, 3, and 5-year intervals were generated, showing high consistency with the expected survival probability, thereby indicating reliable predictive concordance (**Figure 6E**). Additionally, Kaplan-Meier survival analysis was conducted to illustrate the prognostic value of CCR7 and SPP1 in both the training and testing hepatocellular carcinoma (HCC) patient cohorts. The results for the training cohort showed marginal significance for CCR7 (p = 0.07) and significance for SPP1 (p = 0.0033), while in the testing cohort, significance was observed for CCR7 (p = 0.035) and marginal significance for SPP1 (p = 0.073) (**Figure 6F**). The diagnostic performance of the risk score was evaluated using receiver operating characteristic (ROC) analysis. The area under the curve (AUC) values for predicting 1, 3, and 5-years survival in the training cohort were 0.75, 0.70, 0.71, respectively, and in the testing cohort, they were 0.67, 0.75, 0.73, respectively (**Figure 6G**). Moreover, a heatmap depicting the expression levels of specific genes in patients with high and low risk scores in both the training and testing HCC patient cohorts indicated that the expression levels were significantly higher in the high-risk group compared to the low-risk group (**Figure 6H**). Collectively, the regression model, performance evaluations, and survival analysis results suggested that the SPP1 gene may serve as a prognostic marker for HCC.

### Expression validation and immune infiltration of HCC prognostic gene SPP1

**Figure 7:**
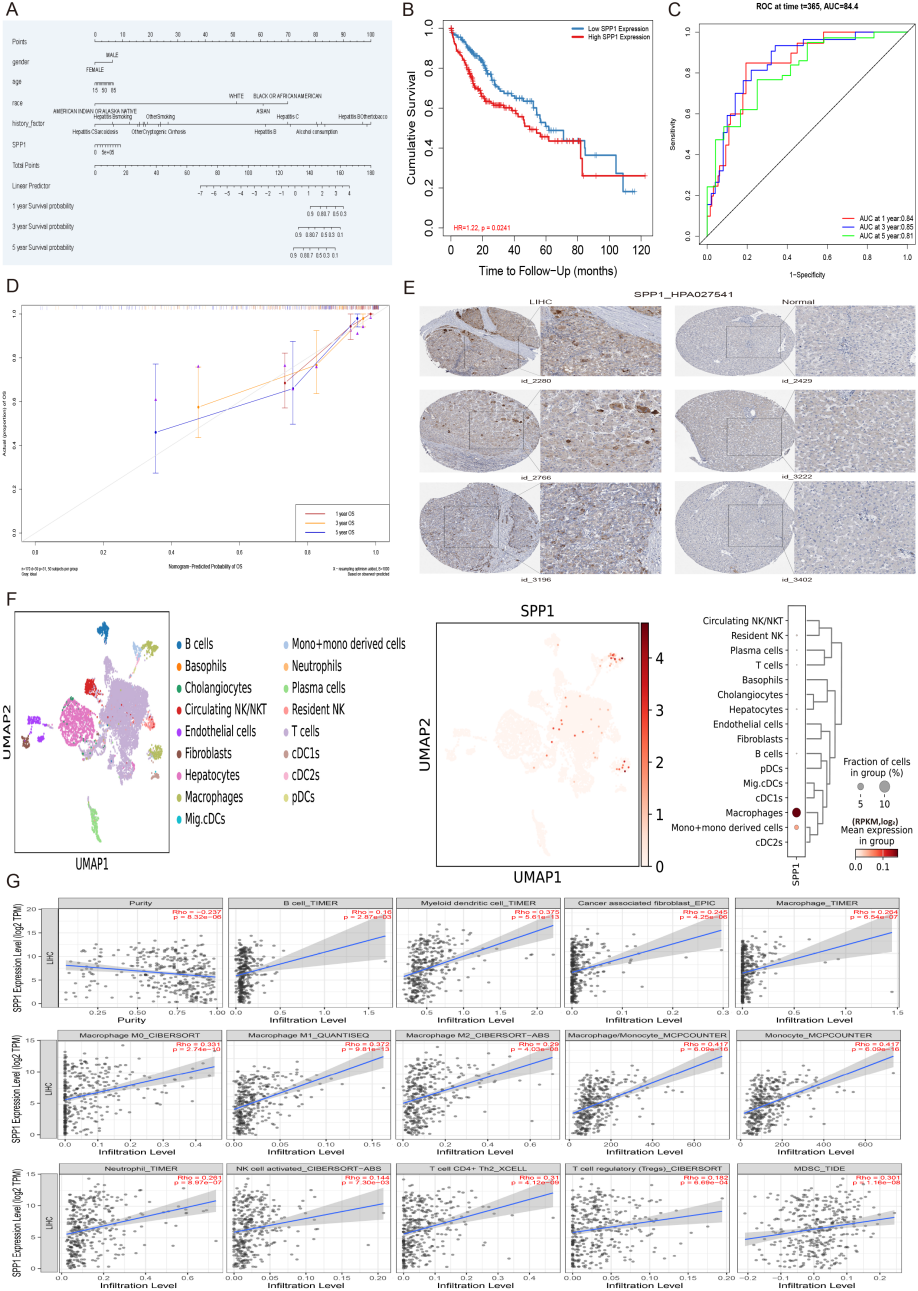
The multivariate analysis, protein expression validation, cell subset expression and immune infiltration of HCC prognostic gene SPP1. **(A)** The nomogram plot of significant multivariate and SPP1 with the 1, 3, and 5-year survival of LIHC. **(B)** The survival analysis of SPP1 gene in the multivariate Cox regression model incorporating age, gender, race, pathological stage factors. **(C)** The receiver operating characteristic (ROC) investigation of model performance, and the area under curve (AUC) values were 0.84, 0.85, and 0.81 for predicting 1, 3, and 5-years, respectively. **(D)** The calibration curves of multivariate Cox regression model for survival at 1, 3, and 5-year intervals displayed high consistency with the expected survival probability. **(E)** The immunohistochemistry results of gene SPP1 in 3 independent hepatocellular carcinoma patient tissues (patient id_2280, patient id_2766, patient id_3196) and 3 independent normal tissues (patient id_2429, patient id_3222, patient id_3402) under the same antibody HPA027541. **(F)** The distribution and expression of prognostic gene SPP1 in cell subpopulations of hepatocellular carcinoma patient tissue samples. **(G)** The immune infiltration analysis of the prognostic gene SPP1 with 14 immune cell subsets in the LIHC, including B cells, cancer-associated fibroblasts, macrophages, macrophages (M0, M1, M2), monocytes, neutrophils, myeloid dendritic cells, macrophages/monocytes, activated NK cells, CD4+Th2 T cells, regulatory T cells (Tregs), and myeloid-derived suppressor cells (MDSCs).

To analyze the effect of SPP1 and other significant multivariate factors on the prognosis of hepatocellular carcinoma (HCC), a multivariate Cox regression model was applied, incorporating gender, age, race and history factors. The results of survival analysis indicated that the SPP1 gene can serve as a prognostic marker for liver cancer (p = 0.0241) (**Figures 7A, B**). Furthermore, to evaluate the performance of the model, the diagnostic performance of the risk score was assessed using receiver operating characteristic (ROC) analysis. The area under the curve (AUC) values were 0.84, 0.85, and 0.81 for predicting 1, 3, and 5- years survival, respectively (**Figure 7C**). The calibration curves for survival at 1, 3, and 5-year intervals displayed high consistency with the expected survival probability, indicating reliable prediction concordance (**Figure 7D**). Based on the results of model validation and risk gene prognostic evaluation, further verification was conducted to examine the expression difference of the prognostic gene SPP1 in HCC patients compared to normal tissues. The protein expression data of HCC patients in the Human Protein Atlas database was analyzed. Immunohistochemistry results showed that the expression level of the SPP1 gene in tissues from three independent HCC patients (patient IDs 2280, 2766, 3196) was significantly higher than that in three independent normal tissues (patient IDs 2429, 3222, 3402), confirming that SPP1 can indeed be used independently as a prognostic marker (**Figure 7E**). To explore the distribution and expression of the prognostic gene SPP1 in cell subpopulations of HCC, single-cell sequencing data from HCC patient tissue samples were analyzed. The visualization results of cell type annotation revealed that the HCC subpopulations were divided into 17 cell subsets, including B cells, Basophils, Cholangiocytes, Circulating NK/NKT, Endothelial cells, Fibroblast, Hepatocytes, Macrophage, Mig.cDCs, Mono+mono derived cells, Neutrophils, Plasma cells, Resident NK, T cells, cDC1s, cDC2s, pDCs. Among these, T cells, Hepatocytes and Macrophages were the main cell subsets. Additionally, the SPP1 gene was highly expressed in Macrophages and Mono+mono derived cells (**Figure 7F**). Therefore, to further investigate the relationship between SPP1 and immune infiltration, computational methods such as TIMER, XCELL, CIBERSORT, CIBERSORTABS, QUANTISEQ, MCP-COUNTER and EPIC were utilized to comprehensively evaluate SPP1 and the immune microenvironment of liver cancer. The analysis results demonstrated that the prognostic gene SPP1 was significantly positively correlated with 14 immune cell subsets in the liver hepatocellular carcinoma (LIHC), including B cells, cancer-associated fibroblasts, macrophages, macrophages (M0, M1, M2), monocytes, neutrophils, myeloid dendritic cells, macrophages/monocytes, activated NK cells, CD4+Th2 T cells, regulatory T cells (Tregs), and myeloid-derived suppressor cells (MDSCs) (**Figure 7G**).

### Association analysis of gene SPP1 with immune infiltration, subtypes, and prognostic development in HCC

**Figure 8:**
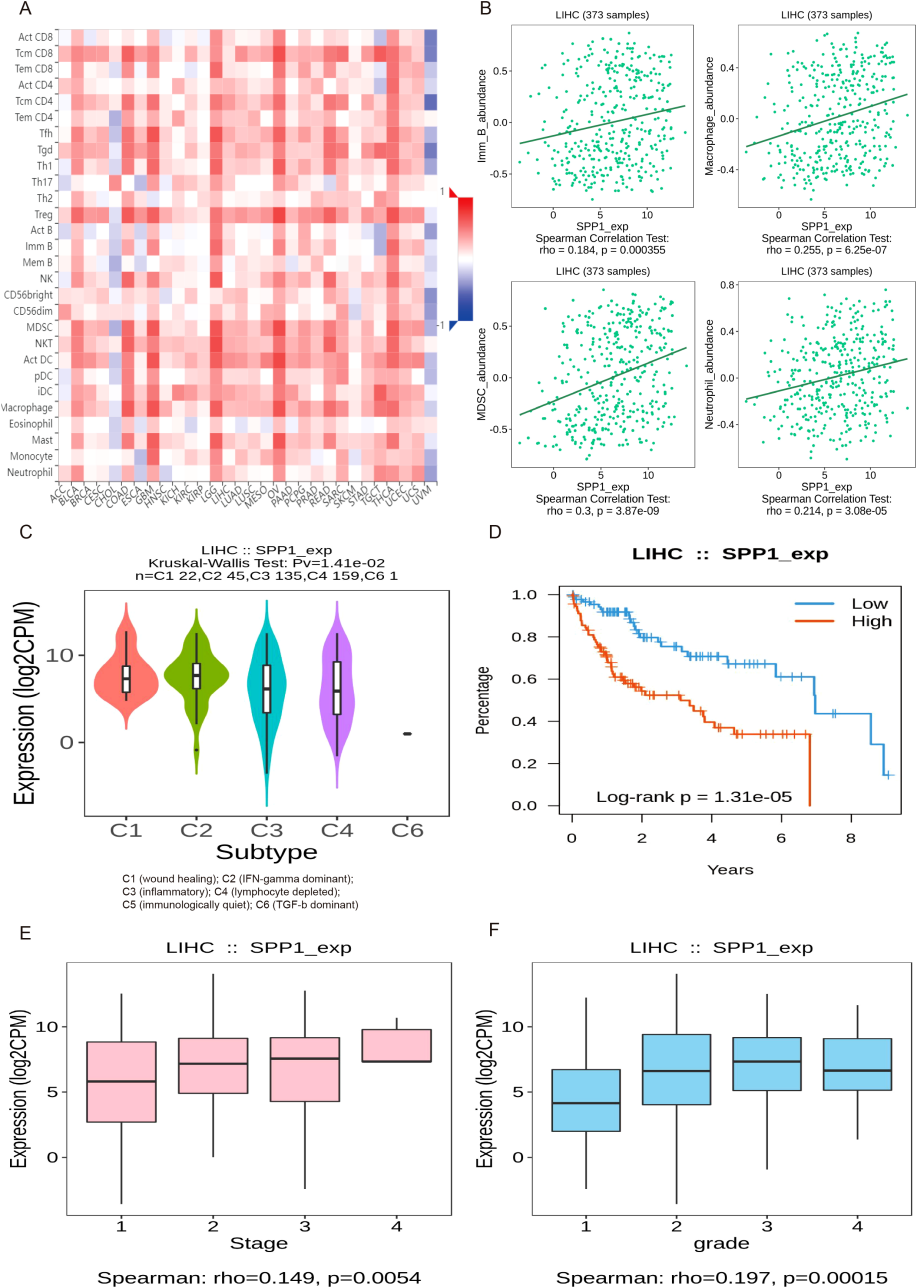
The analysis of the relationship between gene SPP1 expression and immune infiltration, subtypes, and prognostic development in HCC. **(A)** Heatmap of correlation between SPP1 expression and immune cells across human liver cancer. **(B)** Spearman correlations between expression of SPP1 and immune cells in human HCC, including B cells, Macrophages, myeloid-derived suppressor cells (MDSCs) and Neutrophils. **(C–F)** Associations between SPP1 expression and immune subtypes, overall survival, stage and grade in human HCC.

In order to further verify the relationship between SPP1 expression and immune infiltration, an analysis of immune cell correlation in human liver cancer was conducted (**Figure 8A**). The analysis identified four cell types that were positively correlated with SPP1 expression: B cells, macrophages, MDSCs, and neutrophils (**Figure 8B**). Furthermore, an investigation was carried out to examine the relationship between SPP1 expression and immune subtypes, overall survival rate, staging, and grading of liver cancer, aiming to elucidate the connection between SPP1 expression and the prognosis as well as the progression of liver cancer (**Figures 8C–F**). The results of this analysis revealed that SPP1 is primarily closely associated with C3 (inflammatory) and C4 (lymphocyte depletion) immune subtypes. Additionally, high expression of SPP1 was found to be linked to reduced survival time and was also closely correlated with the third stage and grading of HCC.

### Drug discovery of candidate small molecules for liver fibrosis and HCC treatment

**Figure 9:**
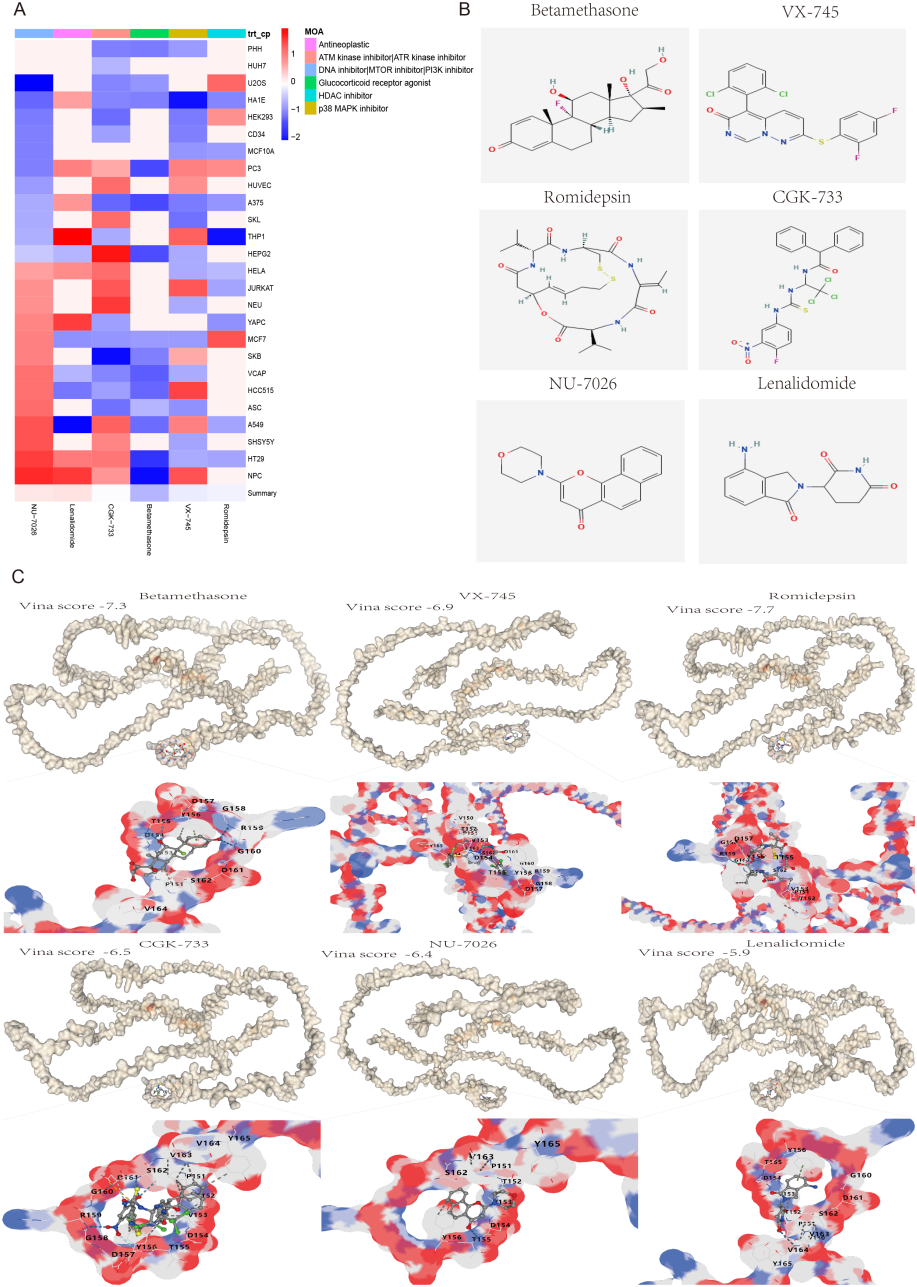
Screening and molecular docking of potential small molecule drugs for the treatment of liver fibrosis and hepatocellular carcinoma. **(A)** Heatmap of the normalized connectivity score (NCS) of 6 small molecule compounds screened from the cMAP database acting on 26 cell types. **(B)** The 2D chemical structures of the six small molecule drugs. **(C)** Molecular docking and Vina score results of potential drugs to their target protein SPP1, including Betamethasone (-7.3), VX-745 (-6.9), Romidepsin (-7.7), CGK-733 (-6.5), NU-7026 (-6.4) and Lenalidomide (-5.9). White represents the protein SPP1, multicolor represents drug binding sites that interact with the protein SPP1.

To further investigate potential small molecule drugs that could exert therapeutic effects in patients with liver fibrosis and hepatocellular carcinoma, 40 highly expressed genes were introduced into the cMAP database as up-regulated genes for prediction of small molecule compounds capable of reversing the expression of pathogenic genes associated with liver fibrosis and HCC. All compounds with an NCS (normalized compound score) less than -2 were filtered out. The NCS results of these screened compounds across 26 cell types revealed that six compounds, namely Betamethasone (a Glucocorticoid receptor agonist), VX-745 (a p38 MAPK inhibitor), Romidepsin (an HDAC inhibitor), CGK-733 (an ATM/ATR kinase inhibitor), NU-7026 (a DNA inhibitor/MTOR/PI3K inhibitor) and Lenalidomide (an antineoplastic agent), exhibited therapeutic potential for the treatment of liver fibrosis and HCC (**Figure 9A**). Furthermore, the 2D chemical structures of these six compounds were illustrated (**Figure 9B**), and protein-ligand molecular docking was performed between SPP1 and these 3D chemical structures (**Figure 9C**). The docking outcomes indicated that the Vina scores of Betamethasone (-7.3), VX-745 (-6.9), Romidepsin (-7.7), CGK-733 (-6.5), NU-7026 (-6.4) and Lenalidomide (-5.9) were obtained, with the Vina score showing a positive correlation with the average NCS value. This suggested that SPP1 possessed good binding ability with these six small molecule drugs and was closely associated with the NCS.

## Discussion

To date, the diagnosis and treatment of liver fibrosis and hepatocellular carcinoma (HCC) still pose significant challenges, partly attributed to the limited understanding of the relationship between liver fibrosis and HCC (45–47). Further research is necessary to precisely elucidate the pathogenic genes and mechanisms at the single-cell level, with the hope of achieving subtype-based targeted therapy for patients with liver fibrosis or HCC and enhancing the understanding of severe liver fibrosis inducing HCC.

In this study, we identified 15 human and 16 mouse cell subpopulations, of which 15 were identical, as well as 60 human and 64 mouse highly expressed genes by analyzing single-cell sequencing data from patients with non-alcoholic steatohepatitis (NASH) and mice with CCl_4_-induced liver fibrosis. These genes may play crucial roles in promoting the development of liver fibrosis. To further investigate the pathogenic mechanism of liver fibrosis, a CCl_4_-induced mouse model of liver fibrosis and bulk sequencing were employed. The results of pathway analysis, gene set enrichment analysis, and immune infiltration analysis not only demonstrated that these differentially expressed genes (DEGs) were closely associated with long-chain fatty acids, triglyceride metabolism and extracellular matrix binding in liver fibrosis but also indicated that various immune cells, particularly macrophages (highly expressing CD163, DOCK8, MERTK and MSR1) (48–51), might participate in the response to liver injury through cell adhesion and actin cytoskeleton regulation signaling pathways, thereby influencing the progression of liver fibrosis. Given that the development of the disease is influenced by the interaction between multiple cells and proteins (52, 53), the results of protein interaction and pathway enrichment analysis of the 40 DEGs revealed that protein interaction network could be divided into three clusters, among which the largest cluster of genes may play a dominant role in the occurrence and progression of liver fibrosis. Furthermore, cell adhesion was significantly upregulated and closely linked to cell activation, signal transduction, and system development, which have been considered to be closely associated with the development of liver fibrosis (54–56).

As mentioned above, liver fibrosis is a chronic liver disease, and its malignant progression ultimately leads to the development of hepatocellular carcinoma (HCC) (57, 58). To further enhance the understanding of the role played by the 40 identified differentially expressed genes (DEGs) in the progression of HCC induced by liver fibrosis, the transcriptome data of 424 patients from the TCGA-LIHC datasets were subjected to differentially analysis using three algorithms (DESeq2, edgeR, and limma). This analysis yielded 1779 identical upregulated genes, including the 40 DEGs. Furthermore, a Cox proportional hazard model and Lasso regression were applied based on these 40 DEGs, leading to 7 specific genes (CCR7, COL3A1, FMNL2, HP, PFN1, SPP1 and TENM4) related to survival outcomes. These genes also belonged to the largest cluster in the protein interaction network, suggesting that they could serve as key genes in the development and construction of a prediction model for liver fibrosis and HCC. Notably, the results of ROC curve and Kaplan-Meier analyses indicated that the SPP1 gene could function as a prognostic marker to accurately predict the prognosis of HCC patients (AUC = 75.5). Patients with high SPP1 expression had significantly shorter overall survival (OS) compared to those with low expression (P=0.0033). Additionally, the nomogram incorporating significant variables and a multivariate Cox regression model, which included age, gender, race, and pathological stage, also demonstrated that the SPP1 gene had a significant impact on the prognosis of HCC (p = 0.0241). These findings were consistent with the results of the Kaplan-Meier analysis of SPP1 in LIHC and were further validated by independent external liver cancer datasets (LIRI-JP) and clinical HCC patient tissue expression data. Meanwhile, the survival calibration curves at 1, 3, and 5-year intervals exhibited high consistency with the expected survival probability. Furthermore, the receiver operating characteristic (ROC) analysis and high- and low-risk expression heatmaps in both the training and testing HCC patient cohorts demonstrated the model’s good diagnostic performance for risk scoring. Therefore, these results not only indicated that the constructed prediction model possessed reliable predictive ability but also demonstrated that SPP1 could indeed serve independently as a prognostic marker for HCC patients.

SPP1, also known as Osteopontin (OPN), is a multifunctional protein and inflammatory cytokine that is widely present in the extracellular matrix, which has been reported to promote the development of liver fibrosis and HCC (59, 60). The tumor microenvironment (TME) is considered to play a crucial role in the occurrence and metastasis of HCC, and relevant studies have only reported the overall characteristics of TME immune cell infiltration (61–63). Our study found that the immune related gene SPP1, as a key prognostic marker for HCC patients, was positively correlated with 14 specific HCC-related immune cells, including B cells, macrophages, MDSCs and neutrophils. These immune cells have been reported to play essential roles in the development of the microenvironment, immune escape and poor prognosis of HCC (64–66). Furthermore, the analysis results of subtypes and prognostic development in HCC also showed that SPP1 is primarily closely related to C3 (inflammatory) and C4 (lymphocyte depletion) subtypes. High expression of SPP1 can lead to reduced survival time, as well as being closely associated with the third stage and grading of HCC. Additionally, some previous research has only reported that molecular heterogeneity is a critical characteristic of tumor or fibrosis occurrence (67, 68). Our research investigated the differences in expression and distribution of SPP1 between liver fibrosis and HCC, and discovered that SPP1 was significantly overexpressed in cholangiocytes in cases of liver fibrosis, but it was highly expressed in macrophages in cases of HCC. These findings suggested that there was cellular heterogeneity in SPP1 gene expression between liver fibrosis and HCC. Furthermore, it was indicated that elevated levels of SPP1 expression could stimulate immune cells, particularly macrophages, to respond to liver injury in the process of liver fibrosis developing into HCC. Overall, the results mentioned above indicated that SPP1, as a prognostic gene or biomarker, was not only significantly negatively correlated with the survival of patients with hepatocellular carcinoma (HCC), but it may also play a pivotal role in promoting the immunosuppressive tumor microenvironment and accelerating the progression of HCC. Consequently, targeting SPP1 and decreasing its expression level could represent an effective therapeutic strategy for patients with liver fibrosis and HCC.

The cMAP is the world’s largest perturbation-based gene expression profile database, capable of unveiling relationships between diseases, genes and drugs. Consequently, an analysis was conducted on the 40 differentially expressed genes (DEGs), leading to the screening of six compounds with normalized compound scores (NCS) less than -2. These compounds were identified as potential small molecule drugs that could reverse liver fibrosis and hepatocellular carcinoma (HCC) gene expression. It is noteworthy that the six screened small molecules possess well-defined mechanisms of action. These include Betamethasone (a Glucocorticoid receptor agonist), VX-745 (a p38 MAPK inhibitor), Romidepsin (an HDAC inhibitor), CGK-733 (an ATM/ATR kinase inhibitor), NU-7026 (a DNA/MTOR/PI3K inhibitor), and Lenalidomide (an Antineoplastic agent). These molecules are either closely linked to the occurrence and progression of cancer or have already been developed into approved clinical drugs. Specifically, Glucocorticoid is known to inhibit neutrophil apoptosis and NF-κB transcription factors. Betamethasone, an approved systemic corticosteroid, binds to the glucocorticoid receptor to suppress pro-inflammatory signals, thereby exerting immunosuppressive and anti-inflammatory effects (69, 70). P38 is a crucial branch of the MAPK pathway, playing a key role in various physiological and pathological processes, including inflammation, apoptosis, cell cycle, and scaffold protein function. VX-745, a highly selective p38α inhibitor, can cross the blood-brain barrier and has been applied in therapeutic trials for Alzheimer’s disease and mild cognitive impairment (71, 72). Romidepsin, an FDA-approved inhibitor of histone deacetylase (HDAC), interacts with zinc ions in the active site of HDAC enzymes to restrain overexpressed HDAC in tumors, restoring normal gene expression and inducing cancer cell apoptosis (73, 74). ATM and ATR are a class of serine/threonine protein kinases considered the main controllers of the cell cycle checkpoint signaling pathway, capable of phosphorylating and activating proteins involved in inhibiting DNA replication and mitosis (75–77). Activation of the PI3K/AKT/mTOR pathway has been reported to promote HCC cell proliferation, migration, and invasion. NU7026, an efficient DNA-PK/PI3K inhibitor, enhances G2/M cycle arrest and apoptosis (78–80). Lenalidomide, an FDA-approved immunomodulatory drug for the treatment of multiple myeloma, marginal zone lymphoma, and follicular lymphoma, exhibits potent antitumor and anti-inflammatory properties (81, 82). The results indicated that the six screened small molecule drugs have significant therapeutic effects on inhibiting inflammation, proliferation, invasion, and promoting cancer cell apoptosis. Furthermore, the Vina scores from molecular docking not only demonstrated good affinity between SPP1 and the compounds (Betamethasone: -8.3, VX-745: -8.1, Romidepsin: -7.9, CGK-733: -6.5, NU-7026: -6.4, and Lenalidomide: -5.9), but also showed a positive correlation with the average value of NCS. Therefore, the six potential small molecule drugs selected for treatment of liver fibrosis and HCC provide crucial insights for future clinical interventions.

## Conclusion

In summary, this study comprehensively analyzed the relationship between liver fibrosis and hepatocellular carcinoma (HCC) by integrating single-cell and bulk sequencing, mouse models, and molecular experiments. It identified 40 pathogenic genes, 15 critical cell subpopulations, as well as cell adhesion and actin cytoskeleton regulatory signaling pathways that promote the development of liver fibrosis and HCC. Furthermore, the study identified and evaluated 7 specific prognostic genes (CCR7, COL3A1, FMNL2, HP, PFN1, SPP1 and TENM4) using a prediction model. It also elucidated the expression heterogeneity of core gene SPP1 and its positive correlation with immune infiltration and the prognostic progression of HCC. Additionally, our study screened six small molecule drugs with high binding affinity and antitumor activity: Betamethasone, VX-745, Romidepsin, CGK-733, NU-7026, and Lenalidomide, providing valuable insights into the prognosis and targeted therapy of liver fibrosis and HCC.

## Data Availability

The data presented in the study are deposited in the NCBI, HPA and TCGA repository, accession number GSE212837, GSE132662, GSE242889, HPA027541 and TCGA-LIHC (https://portal.gdc.cancer.gov/projects/TCGA-LIHC).
